# Optical molecular imaging in oral- and oropharyngeal squamous cell carcinoma using a novel uPAR-targeting near-infrared imaging agent FG001 (ICG-Glu-Glu-AE105): An explorative phase II clinical trial

**DOI:** 10.7150/thno.100042

**Published:** 2025-01-01

**Authors:** Amanda Oester Andersen, Anders Christensen, Karina Straede, Mads Lawaetz, Christoffer Holst Hahn, Nicklas Rubek, Irene Wessel, Giedrius Lelkaitis, Katalin Kiss, Natasja Paaske, Anne Poulsen, Christian von Buchwald, Andreas Kjaer

**Affiliations:** 1 Department of Otorhinolaryngology, Head & Neck Surgery and Audiology, Copenhagen University Hospital, Rigshospitalet, Denmark.; 2 Department of Clinical Physiology, Nuclear Medicine & PET and Cluster for Molecular Imaging (CMI), Copenhagen University Hospital, Rigshospitalet & Department of Biomedical Sciences, University of Copenhagen, Denmark.; 3 Department of Pathology, Copenhagen University Hospital, Rigshospitalet, Denmark.; 4 FluoGuide A/S, Copenhagen, Denmark.

**Keywords:** head and neck cancer, fluorescence-guided surgery, optical imaging, uPAR, near-infrared

## Abstract

**Background**: In oral and oropharyngeal squamous cell carcinoma (OSCC, OPSCC), frequent inadequate surgical margins highlight the importance of precise intraoperative identification and delineation of cancerous tissue for improving patient outcomes.

**Methods**: A prospective, open-label, single-center, single dose, exploratory phase II clinical trial (EudraCT 2022-001361-12) to assess the efficacy of the novel uPAR-targeting near-infrared imaging agent, FG001, for intraoperative detection of OSCC and OPSCC. Macroscopic tumor detection was quantified with sensitivity and intraoperative tumor-to-background ratio (TBR). Microscopic tumor-specificity was assessed by analysis of morphological co-localization between tumor tissue, uPAR-expression, and optical signal. Blood samples were collected up to 44 hours post-injection to further characterize the pharmacokinetic profile of the agent. The trial was conducted with close safety monitoring.

**Results**: Sixteen patients undergoing primary surgical resection were systemically administered 36 mg (n = 4), 16 mg (n = 8), or 4 mg (n = 4) of FG001 the evening prior to surgery. Intraoperatively, using a near-infrared imaging system, real-time optical imaging successfully identified all 16 tumors (sensitivity: 100%, mean TBR: 2.99 range: 2.02 - 3.95), and tumor-specificity was confirmed by histology. Clinical neck metastasis was detected with optical imaging. The maximal plasma concentrations were measured after 1 hour, and the half-life of FG001 was 12 hours. No drug-related or serious adverse events were observed.

**Conclusions**: FG001 holds great potential for optical molecular imaging of OSCC and OPSCC. Further trials are warranted to explore FG001 for intraoperative margin delineation and as a decision-making tool.

## Introduction

Oral and oropharyngeal squamous cell carcinoma (OSCC, OPSCC) are accountable for a significant global disease burden, with 476,100 new cases and 225,900 deaths yearly 1. Surgical resection of tumors and metastases remains a principal therapeutic modality in managing OSCC and OPSCC. The surgical procedure aims to achieve complete removal of cancerous tissue while minimizing functional impairment, including effects on speech, swallowing, and upper airway functions 2,3. The extent of tumor resection thereby inherently implies a delicate balance between achieving tumor-free margins and preserving acceptable functional outcomes. Notably, tumor-positive margins, characterized by viable tumor cells located less than 1 mm from the surgical margin, serve as a well-established predictor for local recurrence and significantly impact disease-specific survival outcomes 4,5. Patients presenting with inadequate margins will normally require re-resection or adjuvant radio-chemotherapy, which due to its toxicities, introduces a substantial risk of short- and long-term treatment-related morbidities 6. Despite this awareness, the reported rate of positive resection margins is 5-43% in oral cancer, and among the highest across surgically treated solid malignant tumors 7,8.

The standard practice for determining margin status involves macroscopic bread loaf sectioning of the resected tumor after formalin fixation, followed by microscopic evaluation of formalin-fixed paraffin-embedded (FFPE) tissue sections by a pathologist 9. The histopathological evaluation process is time-consuming, and normally, the histology report is completed and available for the surgent several days to weeks post-surgery. In case of inadequate margins on final pathology, re-resection may be attempted. Still, re-resections are often technically difficult and associated with an increased risk of morbidity and less favorable survival outcomes compared to primary resections with clear tumor margins 10. Intraoperatively, surgeons rely solely on visual inspection and palpation of tissues to delineate tumors. Frozen sectioning is the only available technique for intraoperative margin assessment, and as a limited number of samples can be processed due to time and costs spent, only a fraction of the interphase between the tumor resection and resection cavity surface can be evaluated 11. Consequently, frozen section sampling is prone to sampling error, especially in large tumor resections, with a reported sensitivity of 10.8% for tumor-positive margins compared to the final histopathological assessment 12. Therefore, there is an unmet clinical need for novel techniques that enable accurate intraoperative margin assessment that can be readily encompassed in the workflow of surgical cancer management.

Optical molecular imaging (OMI) is a promising real-time cancer visualization technology. By applying tumor-specific fluorescent imaging agents and dedicated imaging systems for wide-field or endoscopic near-infrared (NIR) imaging, cancerous tissue can be directly visualized real-time with sufficient contrast to allow fluorescence-guided cancer surgery 13. Over the last decades, numerous candidate imaging agents have been explored preclinically, and recently, a limited number of agents have moved into phase I/II clinical validation, and some phase III trials are ongoing 14,15. With the high resolution and depth of tissue penetration of NIR imaging, OMI has the potential to improve survival outcomes of surgically treated cancer patients by increasing the surgeon's visual feedback during surgery. Targeted OMI may limit the rate of close or involved margins and missed satellite tumor lesions both intraoperatively and by improving the pathological assessment of the tumor specimen postoperatively.

A prerequisite for successfully translating targeted OMI is the detection of cellular targets highly specific to cancer. The urokinase-type Plasminogen Activator Receptor (uPAR) plays a key role in the plasminogen activator (PA) system. It is overexpressed in various types of solid cancers with little or absent expression in normal tissues 16,17. In cancer, the activated PA system is involved in invasion by degradation of the extracellular matrix, angiogenesis, and metastasis, and high levels of uPAR expression are associated with tumor aggressiveness and poor prognosis 18. Across different types of cancers, uPAR is commonly expressed on tumor cells and cellular components of the tumor-associated stroma 19. Thus, using different targeting moieties, uPAR-directed imaging, and therapeutic strategies have been explored clinically in cancer management 20-22. In OSCC and OPSCC, the expression level of uPAR is > 95%, and a high tumor expression pattern has been reported 23-25.

FG001 is an optical agent that binds specifically to uPAR after systemic administration. FG001 consists of the uPAR-binding nonameric peptide AE105, conjugated to the fluorophore indocyanine green (ICG), with two glutamic acid residues as a linker in between (ICG-Glu-Glu-AE105) 26. The high-affinity binding moiety AE105 has also successfully been utilized in the development of uPAR-targeted PET imaging agents where phase II clinical trials in different types of cancer, including head and neck squamous cell carcinoma (HNSCC), have been conducted 22,27-29. ICG is one of the few clinically approved fluorophores in the NIR spectrum (700-1100 nm) with favorable physiochemical- and optical properties and safety profile 30. The biochemical properties of FG001 and the preclinical validation of the tumor-specific visualization of several types of cancers, including OSCC, have previously been reported 26,31,32. The initial clinical validation of FG001 has included a first-in-human open-label nonrandomized phase I/IIa trial, which was a dose escalation (from 1 to 48 mg) design in high-grade malignant gliomas (EudraCT: 2020-003089-38, submitted for publication). Data from the phase I/IIa trial on 40 patients with malignant glioblastoma showed that FG001 was safe and well-tolerated with only 3 grade I, and 1 grade II drug-related adverse events. Tumors could be optically visualized and delineated with acceptable tumor-to-background ratios (TBRs) 15,33.

This phase II trial aimed to explore the efficacy of FG001 for optical visualization of OSCC and OPSCC. The primary endpoint was sensitivity for tumor detection verified by histology. Secondary endpoints were the intraoperative signal intensity assessed as TBR, the pharmacokinetic profile, and the safety & tolerability of FG001. Exploratory endpoints were the microscopic tumor specificity, the ability to detect metastasis, and the characterization of the time window for imaging post-drug injection.

## Methods

### Study design and participants

This prospective, single-arm, open-label, single-center, single dose, exploratory phase II clinical trial investigating the efficacy of FG001, an imaging agent, in patients with OSCC and OPSCC was conducted at the Department of Otorhinolaryngology, Head & Neck Surgery and Audiology, Copenhagen University Hospital, Rigshospitalet, Denmark. The study protocol was approved by the Danish Health and Medicines Authority and the Ethical Committee of the Capital Region of Denmark (Case no.: 2209466) and performed in compliance with Good Clinical Practice (GCP) and the Declaration of Helsinki. This trial was registered at the European Clinical Trials Register: “FG001-CT-003”, EudraCT number: 2022-001361-12.

**Figure 1:**
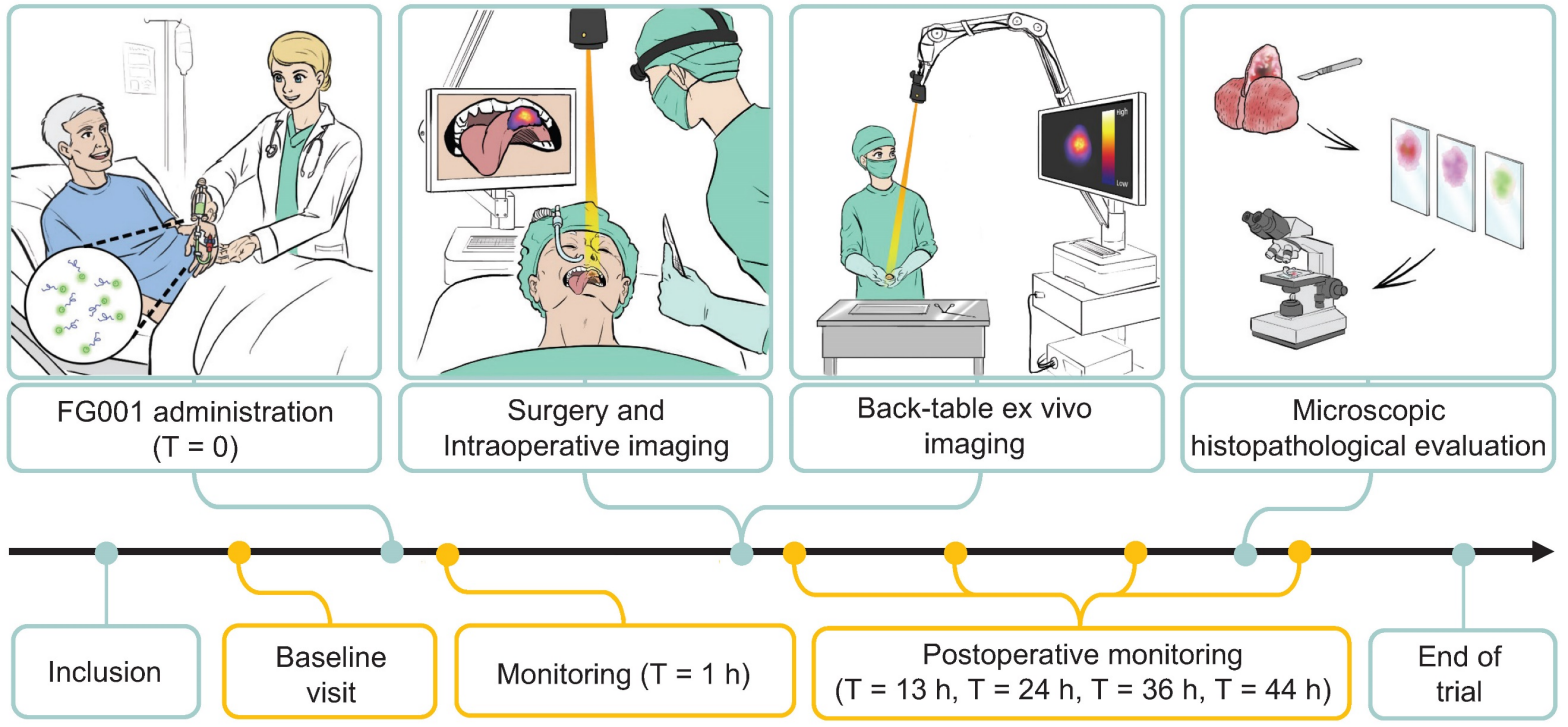
** The clinical trial workflow.** Patients were included and examined at a baseline visit. When meeting all eligibility criteria, FG001 was administered the afternoon prior to surgery (defined as T = 0). Peri- and intraoperative optical imaging was performed in relation to tumor resection. Additionally, back-table *ex vivo* imaging of the tumor specimen was acquired along with a biopsy for sensitivity assessment or tumor cross-sectioning for microscopic histopathological evaluation. Microscopic histopathological evaluation consisted of tumor delineation with H&E stain, immunohistochemical staining for target expression, uPAR, and fluorescence scanning with Typhoon or Odyssey Sa. The study was performed with close safety monitoring. The safety-related patient assessments are shown in yellow. h: hour(s).

Consecutive patients above 18 years of age with a histopathological verified primary OSCC or OPSCC scheduled to undergo surgery with curative intent were included. Exclusion criteria included: 1. Patient scheduled for sentinel node biopsy (SNB) with ICG-based optical tracer, 2. Patients with previous surgery, chemo-, or radiotherapy to the oral cavity, 3. Patients with allergy or hypersensitivity to ICG, 4. Female patients who were pregnant or breastfeeding, 5. Patients with overall performance status or co-morbidity deeming the subject unfitted for participation in the trial judged by the Investigator, and 6. Patients with pre-existing hepatic and/or renal insufficiency (INR > 1.7 and/or eGFR < 45 mL/min/1.73 m^2^). All patients received oral- and written participant information and gave their written informed consent before participating in the study. In Figure 1, the trial workflow is schematically illustrated. After inclusion patients were assessed for participation at the baseline visit. Patients eligible for participation were scheduled for surgery and administered FG001 the afternoon prior to surgery. Peri- and intraoperative optical imaging was obtained along with surgery. Furthermore, back-table imaging of the tumor specimen was performed before microscopic histopathological evaluation. The trial was conducted with close safety monitoring (shown in yellow in Figure 1) - including baseline visit and safety monitoring at T = 1 hr, T = 13 hrs, T = 24 hrs, T = 36 hrs, and T = 44 hrs post-injection.

### The investigational medicinal product, FG001

FG001 (i.e., ICG-Glu-Glu-AE105) was synthesized under Good Manufacturing Practice (GMP) (Polypeptide, Strasbourg, France), and preserved as a lyophilized powder for injection (Halix, Leiden, Netherlands). The glass vials acclimatize to room temperature for a minimum of 15 min prior to administration. The investigational drug FG001 was reconstituted in sterile water, resulting in a 1.0 mg/mL FG001 solution. The solubility of FG001 was monitored by visual inspection. To prevent bleaching, FG001 was handled under a green light and protected from surrounding light when transported. The dose (36 mg, 16 mg, or 4 mg) was administered intravenously at a low flow rate through a peripheral vein catheter (PVC) followed by safety monitoring. The PVC was rinsed with isotonic water both before and after administration. The starting dose of 36 mg was selected based on the previous phase I/IIa study in GBM patients, where the maximum dose of 48 mg was reached.

### Safety monitoring

All patients were safety monitored from the time of consent to 44 hrs post-injection of FG001 with vital signs, electrocardiograms (ECGs), blood samples, AE assessments, and physical examinations following the timeline illustrated in Figure 1. ECGs were taken at baseline, 1 hr ± 15 min, and 44 hrs ± 6 h post-injection. Blood samples (including lever and kidney parameters) were sampled at baseline, 12 hrs ± 4 hrs, and 44 hrs ± 6 hrs post-injection. All laboratory analyses were performed by the Department of Biochemistry at Rigshospitalet. AE assessments were graded according to the National Cancer Institute “Common Terminology Criteria for Adverse Events (CTCAE)” version 5.0. There was no data monitoring committee in this trial. However, a safety evaluation meeting between the investigator (i.e., site), sponsor (i.e., FluoGuide), and medical monitor was held once 4 subjects were administered FG001.

### Pharmacokinetic profiling of FG001

To estimate the pharmacokinetic (PK) profile of FG001, blood samples were collected at 1 hr ± 15 min, 13 hrs ± 2 hrs, 24 hrs ± 4 hrs, 36 hrs ± 4 hrs, and 44 hrs ± 6 hrs post-injection. The blood samples were collected in Vacuette® 3 mL K2E K2EDTA vacutainer blood collection tubes, centrifuged (2500 rpm for 10 min at 4 °C), and the plasma was stored in a -20 °C freezer. Plasma concentrations were measured based on liquid chromatography with tandem mass spectrometric detection (LC-MS/MS) validated according to good laboratory practice (GLP), (LabCorp, Huntingdon, UK). The PK analysis was performed using a non-compartmental analysis method. Nominal sampling times were used for determination of descriptive statistics for FG001 plasma concentrations and for plotting mean concentration-time profiles in WinNonlin. The terminal phase half-life (T_½_) was calculated using terminal phase rate constant, which resulted from linear regression of the terminal linear portion of the log-concentration vs. time curve.

### Standard surgical procedure and optical imaging

All patients were treated surgically according to the national guidelines. The primary investigator (PI) was part of the surgical team in all procedures. A surgical robot system (DaVinci Si, Intuitive) was used for TORS in the two patients with OPSCC. The surgeons were not allowed to resect tissue based on intraoperative detected fluorescence signal but complied with standard of care. Depending on the clinical TNM staging (based on clinical examination, ultrasonography examination of the neck, and MRI or CT scans), either sentinel lymph node biopsy (using 99^m^Tc-nanocoll) or neck dissection was performed for neck management. Post-surgical (chemo)radiation was prescribed according to national guidelines at a multidisciplinary conference. The pre-, intraoperative, and ex-vivo optical images were obtained using the open-field imaging system Elevision® model VS3 Iridium-Visionsense Infrared (IR) Fluorescence, Vision System (Medtronics, USA). Image profile: NIR_highcontrast or NIR_contrast, excitation laser: 805 nm. The Elevision IR system generates real-time optical imaging merged with the color video recording on a viewer screen. The intensity-dependent multi-color NIR overlay was presented in parallel with white-light and NIR grayscale images. The camera head was mounted on a maneuverable arm over the surgical field with sterile draping. The preferred camera-to-tumor distance was 20-40 centimeters, but the system also allows positioning at closer or longer distances to areas of interest. For optimal imaging, all other light sources in the OR were switched off and window blinds were closed. The imaging system itself provides powerful white light illumination of the surgical field when imaging is performed.

**Figure 2:**
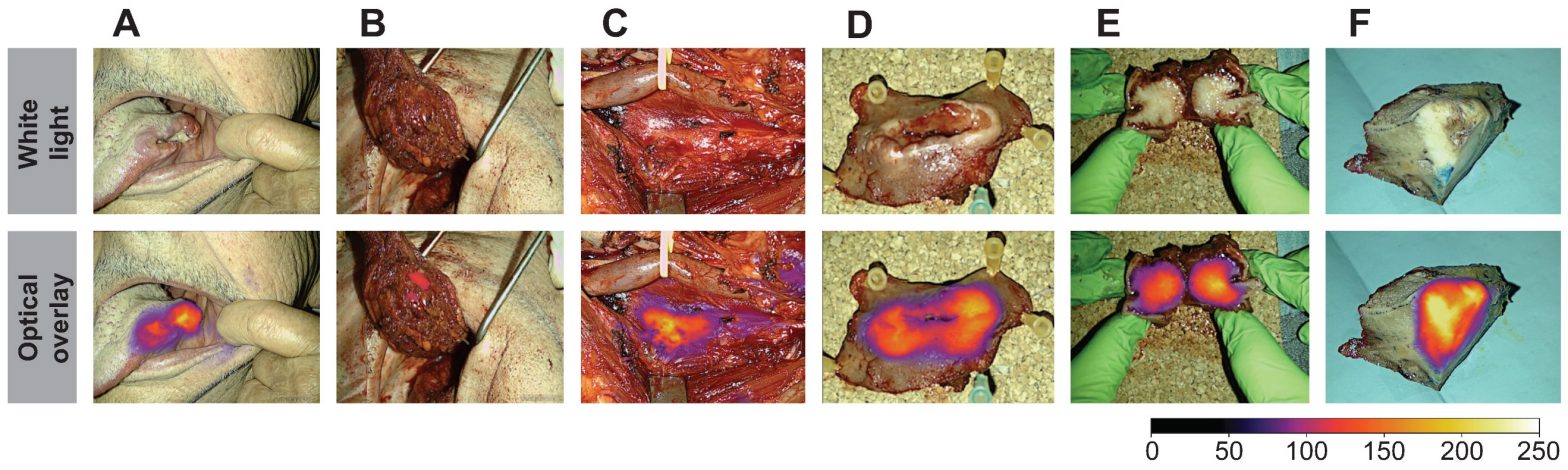
** Peri- and intraoperative images of case 11 shown in white light (top row) and optical overlay (bottom row).** The images were taken with the Elevision NIR camera system.** A:** Preoperative image, **B:** Tumor bed and resection cavity, **C:** Local metastasis, **D:** Back-table imaging of tumor, **E:** Tumor cross-section, **F:** Pathological tumor evaluation after formalin-fixation. Gain: 1%.

All images and videos were stored in JPG- and mp4-file format. The following images were captured for all 16 patients who completed the trial: 1) Preoperative images of the tumor. 2) Intraoperative images of the tumor and metastatic lymph nodes. 3) Images of the surgical cavity (i.e., tumor bed) after tumor resection. 4) Ex-vivo images of the resected tumor specimen on the back-table, see Figure 2.

For imaging analysis, the TBR was calculated as the maximum fluorescence intensity of the tumor region of interest (ROI) divided by the mean fluorescence intensity of 3 background ROIs on a preoperative NIR image by use of an in-house designed Python-based annotation tool. The tumor ROIs were drawn based on information from intraoperative clinical observations, and preoperative white-light and NIR images. Background ROIs were defined within the soft tissues adjacent to the tumor. The absolute numeric intensities were obtained by adjusting the fluorescence intensity for the specific gain values applied by the imaging system.

### Central biopsies based on the fluorescent signal

For 11 out of 16 patients, a single biopsy was sampled from the resected tumor specimen ex-vivo on the back-table from a region with a bright fluorescence presented by the imaging system. For the remaining 5 patients, the tumors were cross-sectioned for detailed optical imaging and histopathological processing (see next section). The back-table imaging was performed with the same settings (Image profile: NIR_highcontrast or NIR_contrast, excitation laser: 805 nm) and with no other light sources, always with a perpendicular angle to the table, and a camera-to-tumor distance of 20 centimeters. The biopsies were taken with biopsy-punch-forceps, hereafter formalin (4%) fixed, paraffin-embedded (i.e. FFPE), and evaluated with histopathological assessment by a pathologist for the presence of cancer tissue - concluding on cancer-positive or negative.

### Cross-sectioning of tumor resections, optical scanning, and immunohistochemical staining

For 5 out of the 16 patients, the resected tumor specimens were cut in half, sliced at the OR, and imaged with the Elevision® imaging system. The specimens underwent routine pathologic processing at the Department of Pathology. The slices were frozen with a Milestone PrestoCHILL cryoembedder/freezer and, cut into 8 µm tissue slices with a Leica® CM1860 Cryostat and placed on glass slides. To evaluate the overlap between fluorescence signal from FG001, uPAR-expression, and tumor tissue, the cryosections were first fluorescently scanned with an Amersham Typhoon^TM^ scanner from Cytiva and, thereafter, stained using immunohistochemistry for uPAR and Cytokeratin (CK), and hematoxylin and eosin (H&E) stained. The Typhoon^TM^ scanner settings were PMT: 350-500 V and excitation wavelength: 785 nm. The immunohistochemical staining for uPAR was performed with the antibody GTX100467 (Gentex, USA), and Cytokeratin with M351529-2 Dako (Agilent). The H&E stain was performed according to standard practice. All tissue slides were scanned with the high-resolution AxioScan 7 (Zeiss, Germany). Some of the FFPE blocs were optically imaged using Odyssey Sa (Li-COR, Biosciences, UK), (laser intensity: between 3-6, focus: 1.8 mm - 3.5 mm).

**Figure 3:**
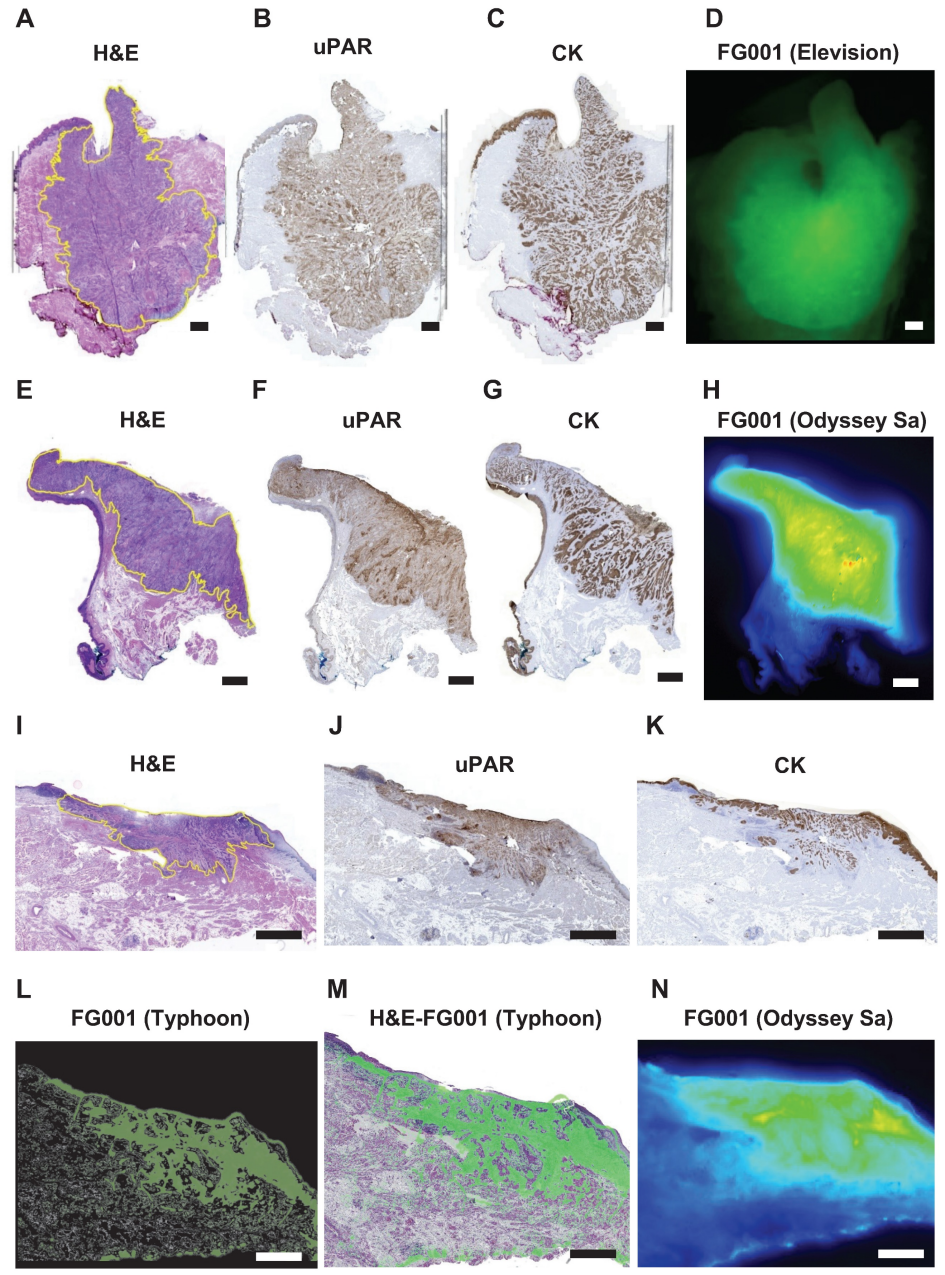
** Microscopic histopathological evaluation of FG001 signal intensity, tumor delineation, and uPAR (target) expression. A-H:** Case 11. **I-N:** Case 15. **A, E, I:** H&E stain. **B, F, J:** IHC uPAR stain. **C, G, K:** IHC cytokeratin (CK) stain. **D:** Optical imaging with Elevision. **H, N:** Optical imaging with Odyssey Sa of FFPE blocks. **L:** Optical Typhoon scan of a fresh frozen 8 μm tissue slice. **M:** Overlay of optical Typhoon scan on the HE stain. Scalebars: 2 mm. IHC: Immunohistochemical. FFPE: formalin-fixed paraffin-embedded.

The different stained tissue sections were aligned using the BigWarp plugin in Fiji (NIH, open-source version: 1.54f) software 34. A pathologist delineated the tumors, and the fluorescence imaging generated from the Typhoon scanner was overlayed on the HE stains using ImageJ (NIH, open source), (see Figure 3M).

### Longitudinal preoperative imaging of the tumors

**Figure 4:**
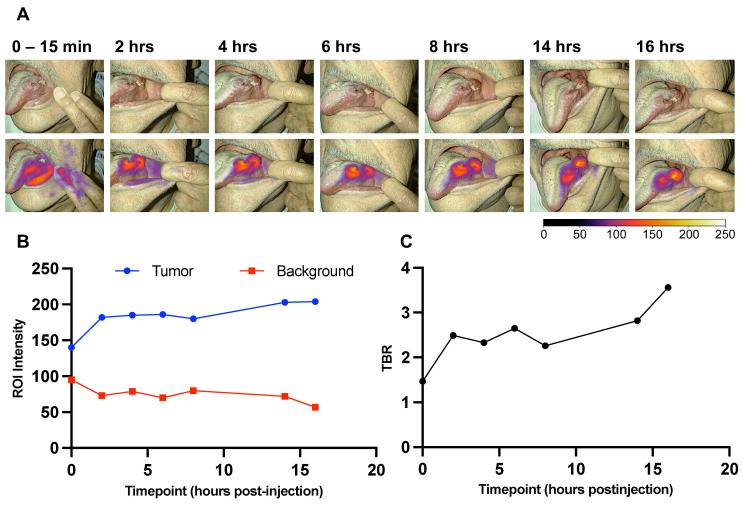
** A longitudinal imaging series of case 11. A:** Images at the indicated timepoints in white-light (upper) and optical overlay (lower). Gain: 1%. **B:** Max ROI intensity of tumor (blue) and mean ROI intensity of background (red) ROI plotted as a function of time. **C:** Tumor-to-background ratio (TBR) advancement over time (0-16 hours). ROI: Region of interest.

For 3 out of the 16 patients, longitudinal preoperative imaging of their tumors was captured during the period before surgery. At the time points: 0-15 min, 2 hrs ± 15 min, 4 hrs ± 15 min, 6 hrs ± 15 min, 8 hrs ± 15 min, 14 hrs ± 15 min, and 16 hrs ± 15 min after injection of FG001, the fluorescence signal was measured with the Elevision® imaging system transorally. Minor deviations from the protocol requirements were due to prioritization of the patients' night sleep before surgery - therefore, no images were taken in the timespan between 8 and 14 hrs post-injection. All longitudinal images were captured with the same camera-to-tumor distance (app. 25-35 cm) in a darkened room. The TBR value was calculated for all time points and plotted as a function of time, see Figure 4 for a case example.

### Trial objectives and endpoints

The main objective of this study was to evaluate FG001 for the detection of OSCC and OPSCC. The secondary objectives were to evaluate the pharmacokinetics of a single i.v. dose and the safety & tolerability of FG001. The primary endpoint was the sensitivity for detection of oral and oropharyngeal cancer verified by histology. The secondary endpoints were divided into efficacy and safety & tolerability. Regarding efficacy, the TBR and intraoperative tumor signal intensity were endpoints. The pharmacokinetics profile determined by a non-compartmental analysis was specified by peak plasma concentration, time of peak plasma concentration, the area under the plasma concentration-time curve from time-zero extrapolated to infinity, and terminal half-life. The safety & tolerability were assessed with adverse event monitoring, laboratory parameters, ECGs, vital signs, and physical examinations.

The exploratory endpoints were defined as: 1) Morphological co-localization between optical signal, tumor tissue, and target (i.e. uPAR) expression, 2) Optical visualization of local neck lymph node metastasis, and 3) Longitudinal investigation of the optimal signal intensity for intraoperative imaging.

### Statistical methods

All clinical-, drug- and imaging-related data were summarized by mean of summary statistics, which were presented as follows: continuous data were presented as mean, and standard deviation (SD), and categorical data were presented as counts and percentages: n (x%). Efficacy was descriptively presented as the proportion of subjects with a positive test result, given they had cancer on pathological evaluation (i.e., sensitivity) for FG001. Due to the novel nature of the intervention, no formal statistical calculation of sample size was deemed reasonable. All statistical analyses were performed in WinNonlin, Python, or Prism 10. A two-sided P value of less than 0.05 was considered significant.

## Results

### Screening and enrollment

**Figure 5:**
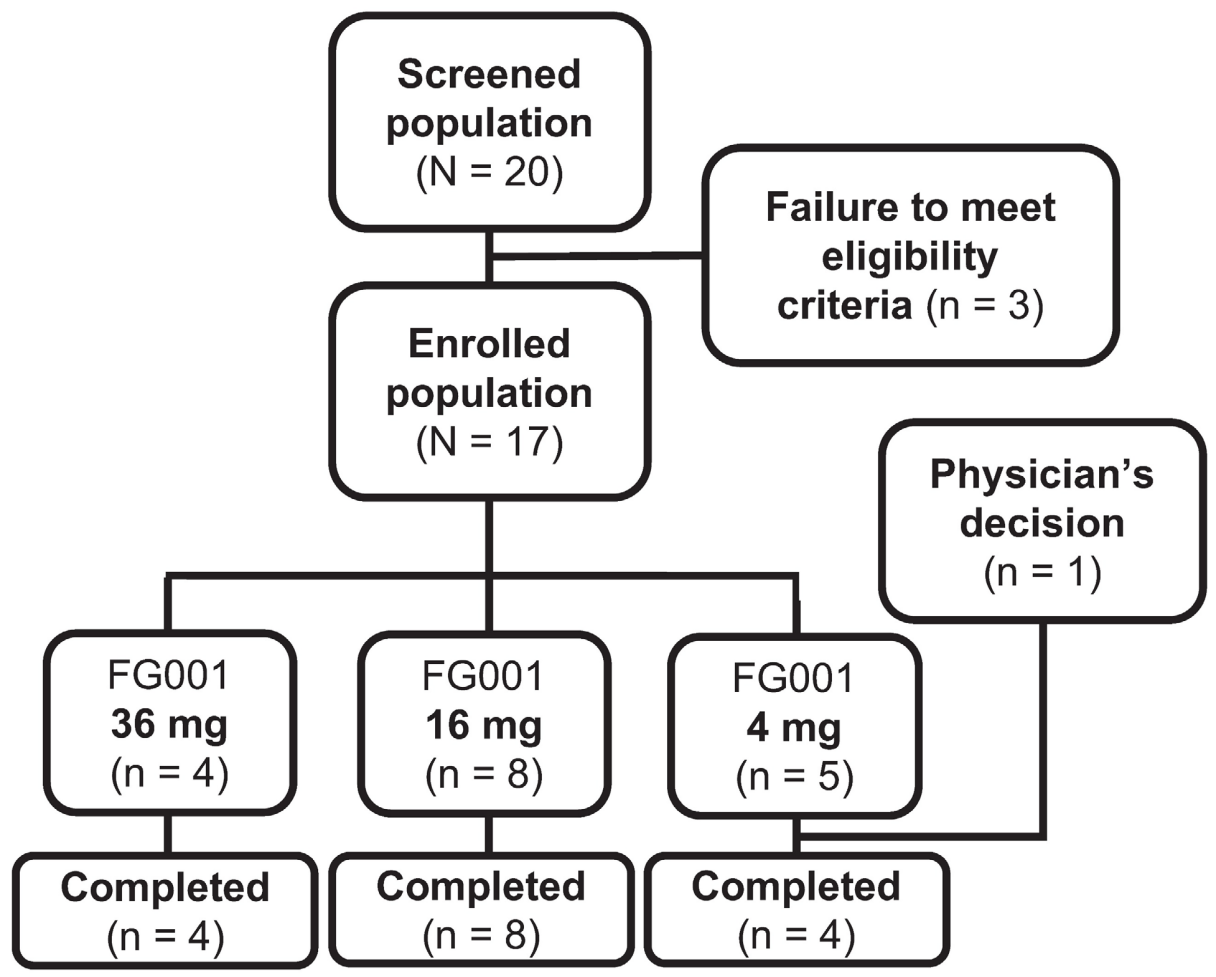
** Patient flowchart from screening to completion. Twenty patients were screened for enrollment.** Three patients failed to meet eligibility criteria (for all exclusion criteria see the methods section): low kidney function defined as eGFR < 45 mL/min/1.73 m2 (n = 1), low lever function defined as INR > 1.7 (n = 1), and previous surgery to T-site (n = 1). The 17 enrolled patients were allocated to the following dose cohorts: 36 mg (n = 4), 16 mg (n = 8), and 4 mg (n = 5). One patient was discontinued by the physicians' decision to omit surgical treatment due to significant growth in tumor size. Resulting in 16 patients completing the trial (36 mg (n = 4), 16 mg (n = 8), and 4 mg (n = 4)).

Between the 18^th^ of November 2022 and the 11^th^ of July 2023, 20 patients with biopsy-verified OSCC or OPSCC were screened for enrolment; three patients were ineligible for participation because of altered kidney- or lever parameters (n = 2) at baseline visit or previous surgery at T-site (n = 1), see Figure 5. The remaining 17 patients were at enrollment allocated to the current dose level group. The patients were systemically administered either 36 mg (n = 4), 16 mg (n = 8), or 4 mg (n = 5) of the investigational medicinal product, FG001. One patient was discontinued after administration of the agent by the physicians' decision to omit surgical treatment due to significant growth in tumor size, resulting in 16 patients completing the trial (i.e., 4 out of 4 in the 36 mg group, 8 out of 8 in the 16 mg group, and 4 out of 5 patients in the 4 mg group).

### Patient demographics and characteristics

In Table 1, an overview of the demographics of the study population is tabulated. The mean age of all 16 patients was 66.7 (range: 29 - 90) years, and body weight ranged from 48.6 to 150.0 kilograms. The primary tumor subsites were the lateral tongue (n = 10), gingiva (n = 2), ventral tongue (n = 1), floor of mouth (n = 1), and oropharynx (i.e., base of tongue, palatine tonsil, n = 2). The subsite lateral tongue was present in all dose cohorts. The two OPSCC patients received 36 mg and 16 mg, respectively. The TNM-stage, depth of invasion (DOI), and UICC8 stage varied across dose cohorts ranging from TNM: T1N0M0 - T4aN2bM0, DOI: 3 - 25 mm, UICC8 stage: I - IVb. The mean depth of invasion was highest in the 16 mg group compared to the 36 and 4 mg groups.

### Tumor detection and intraoperative visualization of OSCC and OPSCC

**Figure 6:**
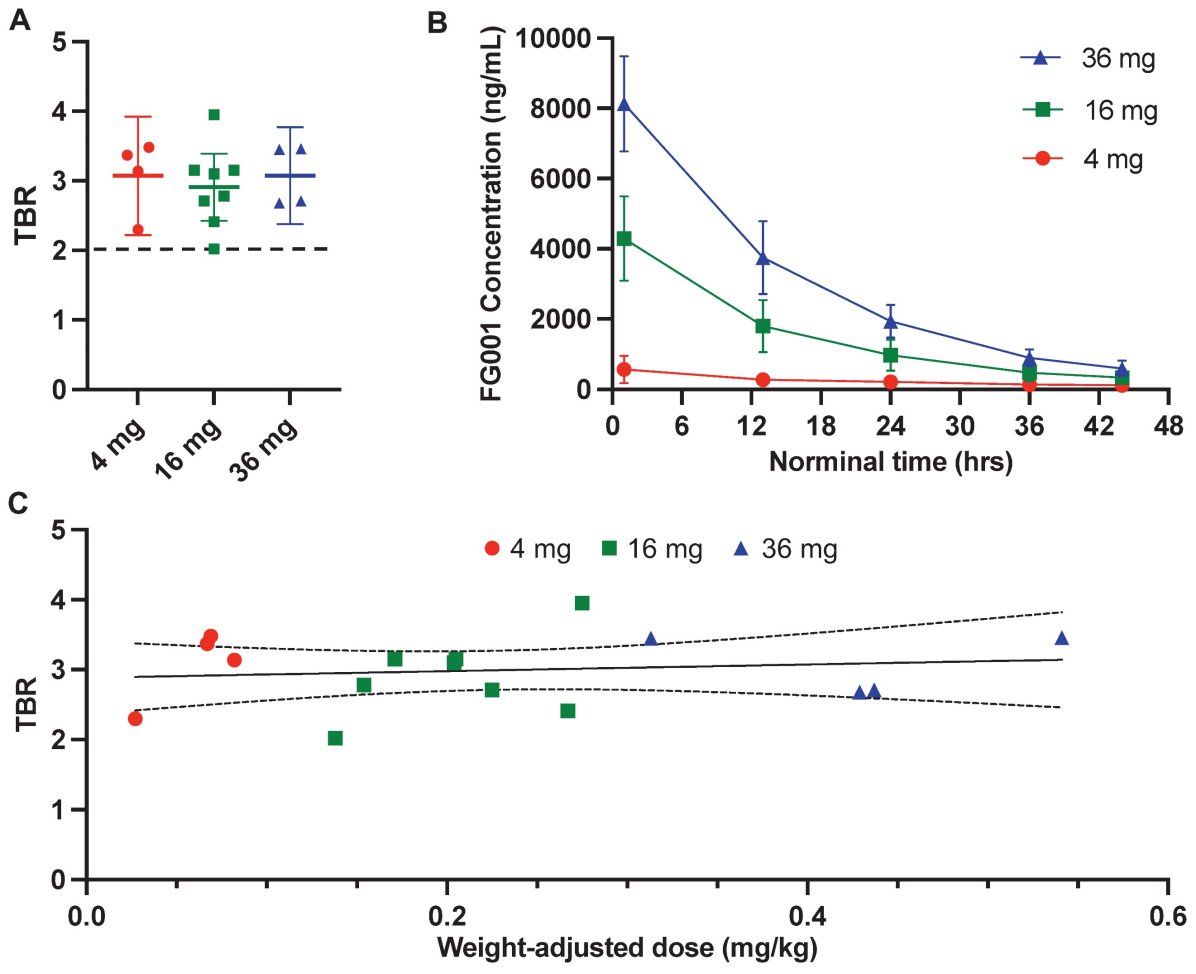
** Quantitative evaluation of the fluorescence signal and plasma concentrations. A:** Mean tumor-to-background ratio (TBR) with 95% confidence intervals (error bars) stratified by dosing group. **B:** Mean (±SD) plasma FG001 concentration modeled as a function of time from injection with stratification by dosing group. **C:** TBR as a function of weight-adjusted dose (mg/kg) with a linear fit and 95% confidence interval. Red: 4 mg, green: 16 mg, blue: 36 mg.

All biopsies sampled by optical guidance and five cross-section samples returned positive for squamous cell carcinoma, resulting in a sensitivity of 100% for tumor detection with FG001. The mean TBRs were not significantly different between the three dose cohorts: 3.08 (SD: 0.44, min-max: 2.68 - 3.46) for the 36 mg group, 2.91 (SD: 0.58, min-max: 2.02 - 3.95) for the 16 mg group, and 3.07 (SD: 0.53, min-max: 2.30 - 3.48) for the 4 mg group (see Figure 6A and Table 2). No positive correlation between weight-adjusted dose and TBR was observed (Figure 6C). When analyzing the absolute intensities, there was an increased tumor- and background signal intensity with increasing FG001 dose at the 3 dose levels corresponding with the plasma concentration measurements (see Table 2). For all 16 patients completing surgery, the mean dosing time from administration to surgery and imaging was 15 hours (range: 12.8 - 19.4 hours). The mean half-life of FG001 was estimated to be approximately 12 hours based on the plasma concentration measurements.

Throughout the surgical procedure, optical imaging of the tumors was performed, including imaging prior to initiation of the tumor resection (Figure 2A), imaging of the tumor bed after resection of the tumor (Figure 2B), and ex-vivo imaging of the tumor specimen on the back table (Figure 2D and 2E). The optical signal from FG001 in resected cancer tissues was preserved after both formalin fixation and paraffin embedding at the Department of Pathology in the days following surgery (Figure 2F & Supplementary information File 1 Figure S1).

**Figure 7:**
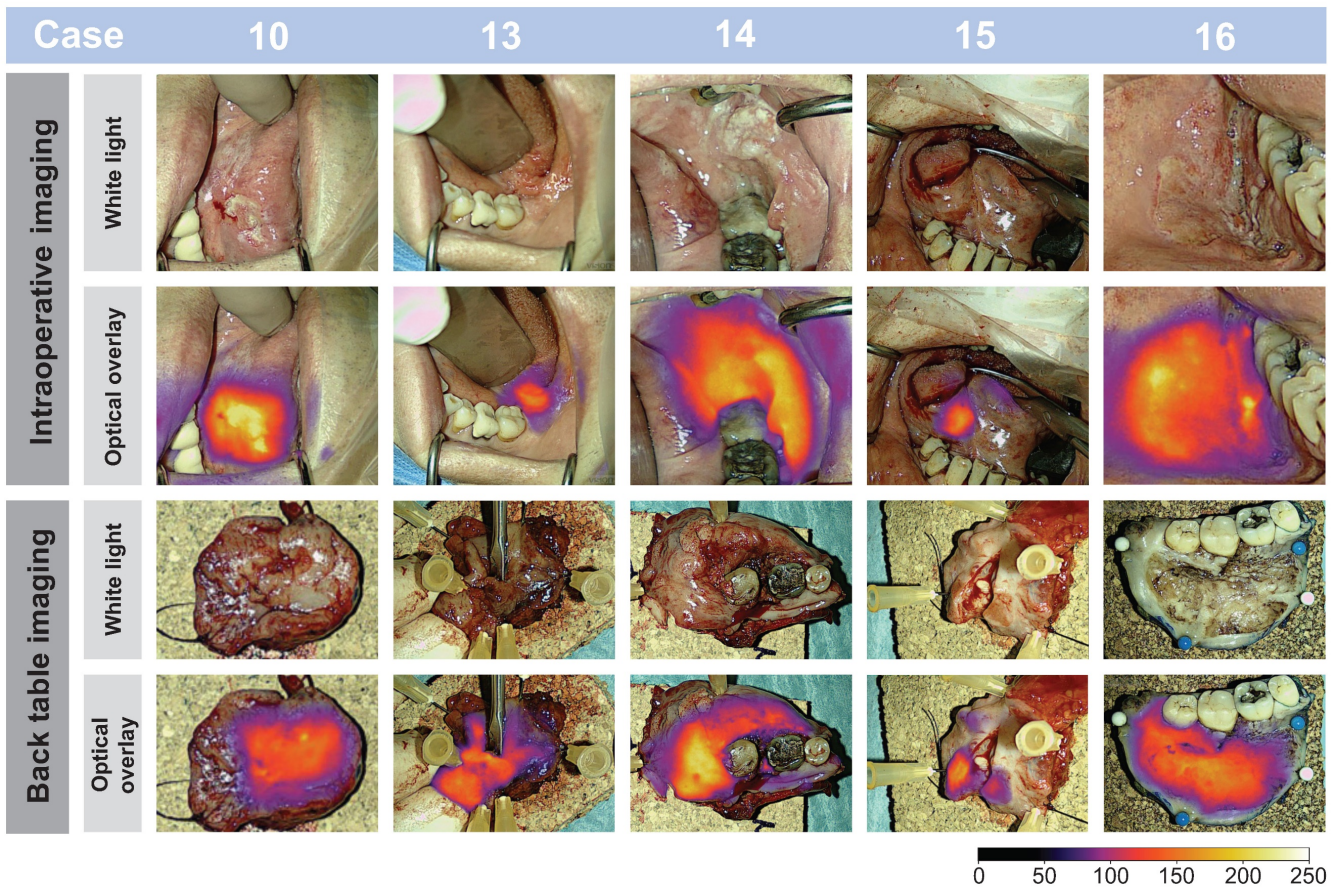
**Real-time intraoperative- and ex-vivo back-table imaging of different patient tumors shown in white light and optical overlay.** Images from case: 10, 13, 14, 15, and 16. Gain: 1 - 5%.

1. Figure 7, a representative subset of tumor images is depicted and shows the imaging possibility of tumors differing in size and location and examples of macroscopic tumor-specific fluorescent signals both in- and ex-vivo. Video footage of tumors *in vivo* is provided, offering insight into the intraoperative performance of the technology in Supplementary Information File 2-3. Intraoperatively, the tumors could be clearly visualized in a wide range of camera-to-tumor distances without affecting the imaging quality remarkedly. The optical delineation of the tumors was notably influenced by the orientation of the camera head, with the most effective imaging achieved when the camera head was positioned perpendicular to the tissue surface. Although imaging for data collection was collected in a darkened operating room (OR), imaging of tumors in ambient light was also possible.

**Figure 8:**
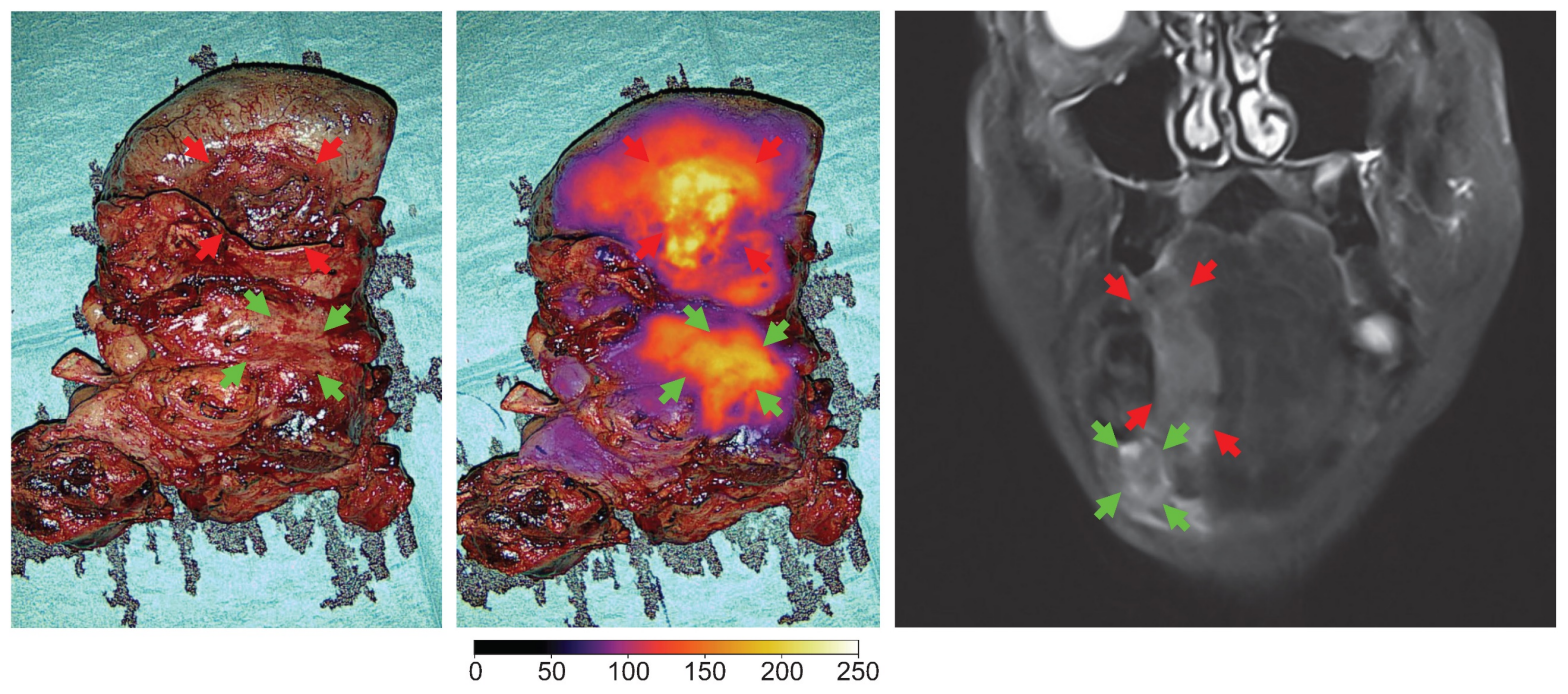
** Optical imaging of the en bloc resection of a FOM/lateral tongue cancer with regional metastasis (level II) in white light and overlay, and the preoperative MRI of the tumor (case 7).** Red arrows: tumor, green arrows: neck metastasis. Gain: 1%.

Case 7 had a large tumor in the floor of mouth with regional neck metastasis (T4N2bM0), where a complex en bloc resection was performed. In this case, optical imaging of the tumor extent and the location of neck metastasis in a large and complicated surgical field was possible (see Figure 8).

### Pharmacokinetic (PK) profile

The plasma concentration of FG001 was measurable in all analyzed samples from the 16 and 36 mg groups; however, it was not determined due to technical issues in some samples in the 4 mg group (i.e., sample at 44 hrs in case 14; 24, 36, and 44 hrs in case 16). Because case 16 did not have three consecutive non-zero concentration measures, the area under the curves (AUCs) could not be determined for this individual. Across all patients with a valid terminal phase, all extrapolations to AUCs from 0 to infinity (AUC_0-inf_) were < 25%.

Following a single systemically administered dose of FG001, the plasma concentrations reached a peak within the first hour post-injection with only one outlier in the 4 mg group (i.e., peaked after 12.8 hrs). The mean and standard deviation (SD) of the maximum plasma concentrations were, mean (SD): 36 mg: 8,130 (1,360) ng/mL, 16 mg: 4,300 (1,200) ng/mL, and 4 mg: 577 (373) ng/mL (Table 2). After the peak, the plasma FG001 concentration declined in a monoexponential fashion, as shown in Figure 6B.

In the 16 and 36 mg groups, the mean (range) of the terminal half-life was 12.9 (11.1 to 13.4) hrs and 11.2 (9.6 to 12.8) hrs, respectively (Table 2). However, there were insufficient data from patients in the 4 mg group and one patient in the 16 mg group (case 8), to determine terminal half-life and other terminal phase-dependent PK parameters.

### Safety and tolerability

During this trial, no drug-related AEs or Serious AEs (SAEs) were registered, see Table 3. A total of 36 Treatment-Emergent AEs (TEAEs) were found, of which 24 were mild (grade 1), nine were moderate (grade 2), and three were severe (grade 3). The registered TEAEs are listed in Supplementary Information File 1 Table S1 - S2, and the most common TEATs were hypoalbuminemia and blood bilirubin increase. No ECG abnormalities were found during the trial. No abnormal findings related to the administration of FG001 were encountered with the physical examinations during the trial.

### Morphological co-localization between FG001 signal, tumor, and target (uPAR-expression)

The resected tumor specimen underwent routine pathologic processing. Tumor cross-sectioning of the tumor specimen in the OR was performed for 5 out of 16 patients across the 3 dose cohorts. The high level of co-localization between the FG001 signal, extent of the tumor, and target expression is shown in Figure 3. The uPAR-expression was observed within the tumor compartment with little or absent expression in the adjacent non-cancerous tissues (Figure 3B, 3F, 3J). The cytokeratin (CK)-expression corresponded to the uPAR-expression within the tumor compartment (Figure 3C, 3G, 3K). The microscopic fluorescence imaging of FG001 showed a high level of co-localization between the uPAR-expression and FG001 fluorescence signal. Additionally, selected formalin-fixed tumor specimens were imaged alongside the pathological bread loaf processing in the days following surgery. The optical tracer signal was preserved throughout the extent of the tumors with a sharp demarcation from the adjacent normal tissues (Figure 3H and 3N).

### Optical visualization of neck lymph node metastasis

**Figure 9:**
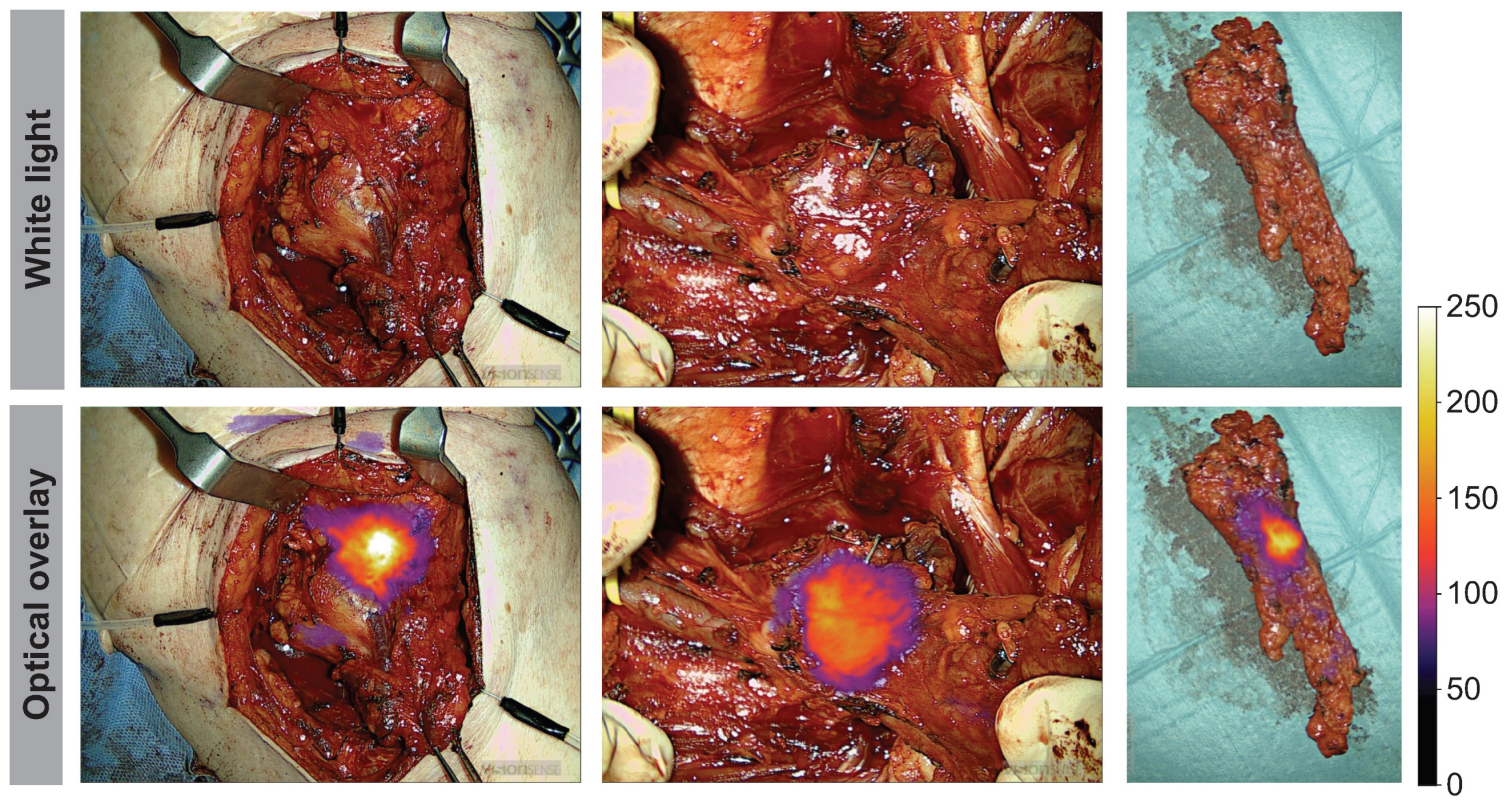
** Real-time intraoperative imaging of neck metastasis and ex-vivo back-table imaging shown in white light and optical overlay.** Gain: 1 - 4%.

Clinical neck metastasis could be clearly visualized intraoperatively during neck dissection by optical imaging, confirmed on the back table by imaging of the resected neck dissection specimen and followed by final evaluation at the Department of Pathology after formalin fixation. Examples are shown in Figure 9. No uptake was observed in adjacent unaffected lymph nodes. However, no systematic optical imaging of lymph nodes without clinical evidence of metastasis was conducted.

In two cases, SNB staging of the neck was undertaken to detect possible occult regional metastatic spread, and optical imaging of resected sentinel nodes was performed. The harvested sentinel nodes had an absent or weak optical signal and were all negative for the presence of subclinical metastatic deposits on final pathology.

### Longitudinal imaging analysis

Longitudinal imaging of tumors on awake patients was performed transorally in three patients (all in the 16 mg dose cohort) prior to surgery. The tumor-specific signals could be detected after 2 hrs post-injection with sufficient TBRs above 2 until 19 hrs post-injection. Furthermore, the signal appeared to be robust over the time of surgery. An example of longitudinal case data (i.e., imaging series and TBR as a function of time) is shown in Figure 4A and 4C. There was a tendency for an increase in tumor intensity followed by a plateau while the background intensity slowly decreased after a fast maximum intensity (Figure 4B).

## Discussion

The major findings from this exploratory phase II trial on FG001, was that a novel uPAR-targeting NIR imaging agent allows tumor-specific and safe real-time in-vivo and ex-vivo imaging of oral and oropharyngeal cancer. FG001 detected tumor-positive tissue with 100% sensitivity. The contrast for demarcation of tumors, expressed as TBR, was consistently above 2, even at the lowest dose, which is accepted as a relevant threshold for clinically applicable optical imaging agents. The longitudinal data for tumor imaging indicate a flexible window for drug administration (i.e., 2 - 19 hrs) prior to surgery - which may readily be incorporated into the clinical workflow. Morphological microscopic histopathological evaluation showed a co-localization between cancerous tissue, uPAR expression, and optical signal as proof of the tumor-specific performance of the imaging agent. As neck metastasis could also be optically visualized, FG001 may have clinical benefits for imaging of both the primary tumor and metastatic disease. Consequently, data from this study is promising and motivates further investigation of FG001 as an intraoperative tool for detection and delineation of cancer with the overall aim of aiding complete removal of cancerous tissue in OSCC and OPSCC patients.

### Margin assessment with optical imaging

The use of tumor-targeted OMI addresses the major challenge in oncological surgery, namely, how to provide accurate intraoperative information on the certainty of complete removal of all cancerous tissue 11. Clearly, the current use of frozen sections of small tissue samples for intraoperative guidance is a sampling technique that only allows access to a fraction of the entire tumor border, which translates into limited accuracy. Also, when a positive margin is encountered on a frozen section, finding back to the exact anatomical location of the positive margin in the wound bed to perform a re-resection is technically challenging and introduces a risk of sampling error 10. In contrast, as demonstrated in this study (Figure 2), OMI allows real-time wide-field high-resolution imaging of entire tumor surfaces and thereby may provide rapid valuable feedback on insufficient tumor margins that can be corrected by re-resection immediately. In addition, direct optical inspection of the entire wound bed can be performed in search of possible residual cancer tissue in case of a non-radical tumor resection. It is still undetermined if the most optimal and accurate use of this technique is direct intraoperative guidance of the tumor resection or back-table imaging of the tumor specimen in an optically controlled and standardized closed-field imaging system 35. Of note, there is an important technical difference between imaging of superficial margins presented directly on the visible mucosal surface and imaging of the deep margins where a rim of normal tissue is overlying the tumor border and creates photon scattering and a degree of attenuation of the optical signal.

From the clinical studies of the antibody-based agents (i.e., cetuximab-800CW, panitumumab-IRDye800) targeting EGFR in head and neck cancers, a major focus has been on margin assessment in closed-field imaging systems on the back-table or during the pathological processing postoperatively. Two studies reported a detailed analysis of the deep and superficial margins of resected tumor specimens from patients injected with panitumumab-IRDye800 and found a high level of co-localization between fluorescence intensity and the location of the tumor 36,37. Van Keulen *et al.* reported a 95% sensitivity and 89% specificity for the detection of inadequate margins (< 5mm) with microscopic histopathology as a reference. They also reported a maximum penetration depth in human tissue of the agent of 6.3 mm. Also, in 18 OSCC patients injected with panitumumab-IRDye800, detection of the closest margin by gross examination of the specimen by the surgeon or optical imaging with a closed-field imaging system, optical guidance had the highest correlation when compared with final pathology 38. De Wit *et al.* studied optical assessment of both superficial and deep margins in a closed-field imaging setting on resected specimens from 65 patients with OSCC injected with cetuximab-800CW and found a sensitivity and specificity of 100% and 85.9% for detection of positive margins (with the threshold: signal-to-background ratio (SBR) 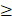
2) and a sensitivity and specificity of 70.3% and 76.1% for detection of close margins (<5mm) (with the threshold: SBR 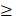
1.5) 39. Accordingly, based on existing data, it appears feasible to determine margin status based on optical signal intensity with an acceptable diagnostic accuracy on both superficial and deep margins. Precise detection of involved margins seems most reliable, while the determination of close margins may be more challenging because the signal attenuation due to non-cancerous tissues overlying the tumor surface must be incorporated in the determination cut-off values for clear margins (≥5 mm). As this current study was a phase II trial with an explorative scope, an analysis of the diagnostic performance of FG001 for detection of positive or close margins by use of a closed-field imaging system was not incorporated but will be investigated in future clinical trials. However, from preclinical data in animal models, a very low threshold of FG001 for detection of tumor deposits in the micrometer range was observed, which is a prerequisite for an optical imaging agent to allow accurate high-resolution tumor margin assessment 40.

### Detection of metastatic lymph nodes using optical imaging

Detection and resection of lymph node metastasis is another aspect of surgical cancer management where targeted optical imaging may have the potential to impact the survival outcomes significantly. In HNSCC, the presence of neck metastasis is the single most important prognostic factor for outcome, and the intended complete removal of regional metastatic disease by neck dissection is a key element of treatment. As demonstrated in this study, OMI aided by FG001, allowed direct wide-field real-time imaging of a large anatomically complicated surgical field to detect focal metastatic lesions embedded in the fibrofatty tissues (Figure 8 - 9). Therefore, targeted optical agents may be an intraoperative tool for localizing metastatic deposits unintentionally left behind in a surgical field with a possible impact on treatment outcome or provide optical guidance in the histopathological workup by localizing metastasis in lymphadenectomy specimens. Interestingly, it was reported that injection of panitumumab-IRDye800 in OSCC patients enabled localization of both sentinel and metastatic lymph nodes with acceptable accuracy and with possible implications for both SNB staging and neck dissection 41,42. Similarly, in a study of 12 HNSCC patients injected with cetuximab-IRDye800CW, postoperative optical imaging of neck dissection specimens in a closed-imaging device showed high diagnostic performance for detection of metastasis with a sensitivity of 97.2%, and in 2 cases neck staging was altered due to additional small metastatic deposits localized by optical imaging 43.

### Peptide-based targeted optical imaging and receptor specificity

The targeting moiety of FG001 is a peptide, distinguishing it from numerous other antibody-based optical imaging agents under current clinical phase I-III testing. Theoretically, this should result in a lower rate of drug-related adverse events, as supported by the findings in this study (Table 3), because peptides have a lower immunogenicity and toxicity than antibodies 44. Indeed, previous clinical trials investigating antibody-based optical targeting agents in head and neck, and other types of cancers did report drug-related both adverse- and severe adverse events, which may have implications on clinical implementation 37,45,46. Furthermore, the rapid plasma clearance of peptides compared to antibodies enables a faster imaging timepoint from several days (antibodies) to hours (peptides) post-injection (Figure 4, Figure 6B), which would be advantageous for the logistics and planning of surgery in a real-world clinical setting.

In this study both macroscopic and microscopic tumor-specific optical imaging by use of FG001 was observed, indicating a receptor-specific binding of the agent (Figure 2 - 3, 7). Furthermore, no dose-TBR relationship was observed in the tested dose interval (i.e., 4 to 36 mg, Figure 6A and 6C), as TBR appeared stable in the investigated dose range. However, a dose-intensity relationship was observed when analyzing the absolute tumor and background intensities (Table 2). Based on these findings, receptor saturation in the investigated dose interval is not likely.

### Endoscopic optical imaging and integration in robotic surgery

Optical imaging of pharyngeal tumors has received limited attention in previous literature, likely due to the need for suitable hardware for successful clinical implementation. In contrast to oral cavity tumors, where open-field systems can be used, pharyngeal tumors require endoscopic or, ideally, robot-integrated imaging systems. As transoral robotic surgery (TORS) has, in the last decades, been widely implemented for surgical resection of OPSCC, integration of optical guidance with targeted agents to improve control of margins seems logical. Importantly, the most used robotic system for TORS (i.e., DaVinci, Intuitive) has an integrated optical imaging system optimized for NIR imaging of ICG 47. Based on experience with optical imaging during TORS in this study, it was observed that proper angling possibilities of the NIR camera head in relation to the tumor surface are crucial for optimal implementation and maximal benefit of the technology, which otherwise appears highly promising.

### Study limitations

The limitation of this study included a small sample size, which limited robust statistical analysis. Further, due to its exploratory nature, an analysis of the impact on determining tumor margin status was not incorporated, nor did the study assess possible clinical benefits from the use of FG001.

## Conclusion

In conclusion, FG001 was demonstrated to be a safe and efficient imaging agent for intraoperative tumor detection in patients with OSCC and OPSCC. The findings from this exploratory phase II trial serve as a proof-of-concept in head and neck cancer, providing impetus for additional clinical trials to investigate the potential clinical application advantages of this uPAR-targeting OMI agent in the surgical management of oral and oropharyngeal cancer.

## Supplementary Material

Supplementary figure, tables, video legends:

Supplementary case 10 video:

Supplementary case 16 video:

## Figures and Tables

**Table 1:** Overview of case specific clinical details

Case no.	Age (years)	Sex (F/M)	Body weight (kg)	Tumor subsite	pTNM	Depth of invasion (mm)	UICC8 stage
**Dose cohort: 36 mg**							
**1**	29	M	84.0	Lateral tongue	T3N2bM0	7	IVb
**2**	60	M	115.0	Lateral tongue	T3N0M0	12	IIIa
**3**	74	M	66.5	Base of tongue (OPC)	T1N0M0	3	I
**4**	73	F	82.4	Lateral tongue	T3N0M0	9	IIIa
**Dose cohort: 16 mg**							
**5**	49	F	58.1	Lateral tongue	T3N0M0	8	IIIa
**6**	78	F	60.0	Lateral tongue	T2N0M0	6	II
**7**	82	M	71.0	Lateral tongue	T4aN2bM0	25	IVb
**8**	80	F	104.0	Floor of mouth	T2N1M0	5	IIIb
**9**	70	M	116.0	Lateral tongue	T2N3BM0	4	IVb
**10**	68	M	78.0	Lateral tongue	T2N0M0	8	II
**11**	74	M	78.6	Lateral tongue	T3N2cM0	19	IVb
**12**	54	M	93.6	Palatine tonsil (OPC)	T3N0M0	17	III
**Dose cohort: 4 mg**							
**13**	66	F	59.9	Lateral tongue	T2N0M0	6	II
**14**	90	F	48.6	Gingiva (Lower gum)	T2N0M0	9	II
**15**	70	F	58.0	Ventral tongue	T2N1M0	4	IIIb
**16**	50	M	150.0	Gingiva (Lower gum)	T3N1M0	6	IIIb

F: female, M: male, OPC: oropharynx cancer. Only completed cases are listed in the table.

**Table 2:** Imaging- and pharmacokinetic parameters

FG001 dose	36 mg (n = 4)	16 mg (n = 8)	4 mg (n = 5)	Total (N = 17)
				
**Weight-adjusted dose** mg/kg (mean (SD))	0.43 (0.09)	0.21 (0.05)	0.06 (0.02)	NA
**Dose timing** hours (mean (range))	14.7 (13.0 - 18.5)	16.0 (12.8 - 19.4)	13.4 (13.1 - 14.1)	15.0 (12.8 - 19.4)
**TBR** (mean (range))	3.08 (2.68 - 3.46)	2.91 (2.02 - 3.95)	3.07 (2.30 - 3.48)*	2.99 (2.02 - 3.95)*
**Absolute tumor intensity** AU (mean (SD))	55.2 (12.4)	30.8 (19.2)	7.9 (5.3)*	NA
**Absolute background intensity** AU (mean (SD))	18.3 (4.9)	11.2 (7.7)	2.5 (1.4)*	NA
				
**Maximum concentration** ng/mL (mean (SD))	8,130 (1,360)	4,300 (1,200)	577 (373)*	NA
**Half-life (T_½_)** hours (mean (range))	11.2 (9.6 - 12.8)	12.3 (11.1 - 15.7)	NA	NA

NA: Not available. Half-life was impossible to calculate in the 4 mg cohort because of technical issues. *: The value is calculated based on data from the 16 patients who completed the trial (i.e., n = 4 in the 4 mg cohort). TBR: tumor-to-background ratio. SD: standard deviation. AU: arbitrary unit.

**Table 3:** Adverse event (AE) monitoring

No. patients (%) / No. events	FG001 36 mg (n = 4)	FG001 16 mg (n = 8)	FG001 4 mg (n = 5)	Total (N = 17)
**TEAE**	4 (100%) / 14	8 (100%) / 15	4 (80%) / 7	16 (94.1%) / 36
**FG001 related TEAEs**	0 (0%) / 0	0 (0%) / 0	0 (0%) / 0	0 (0%) / 0
				
**Mild (Grade 1)**	1 (25.0%) / 8	4 (50.0%) / 11	2 (40.0%) / 5	7 (41.2%) / 24
**Moderate (Grade 2)**	2 (50.0%) / 3	4 (50.0%) / 4	2 (40.0%) / 2	8 (47.1%) / 9
**Severe (Grade 3)**	1 (25.0%) / 3	0 (0.0%) / 0	0 (0.0%) / 0	1 (5.9%) / 3
**SAEs**	0 (0%) / 0	0 (0%) / 0	0 (0%) / 0	0 (0%) / 0

TEAE: Treatment Emergent Adverse Events, SAEs: Serious Adverse Events. Graded according to the Common Terminology Criteria for Adverse Events (CTCAE) v5.0, 27^th^ Nov. 2017.
