# Supplementary material for: Optical molecular imaging in oral- and oropharyngeal squamous cell carcinoma using a novel uPAR-targeting near-infrared imaging agent FG001 (ICG-Glu-Glu-AE105): An explorative phase II clinical trial
Source: Theranostics. 2025 Jan 1;15(1):52–67. doi: 10.7150/thno.100042 (PMC11667226; doi:10.7150/thno.100042)
Supplement: Supplementary file 1 — Supplementary figure, tables, video legends: [file thnov15p0052s1.pdf]

Supplementary information:

File 1, Figure S1: Tumor evaluation after formalin fixation at the Department of Pathology

File 1, Table S1: Treatment-Emergent Adverse Events (TEAEs) registered during the trial

File 1, Table S2: TEAEs by Primary System Organ Class and Preferred Term

File 2: Video footage of tumors in vivo (case 10), filename: *case 10 video.mp4*

File 3: Video footage of tumors in vivo (case 16), filename: *case 16 video.mp4*

File 1:

Figure S1: Tumor evaluation after formalin fixation at the Department of Pathology (white-light and optical overlay). Gain: 9%.

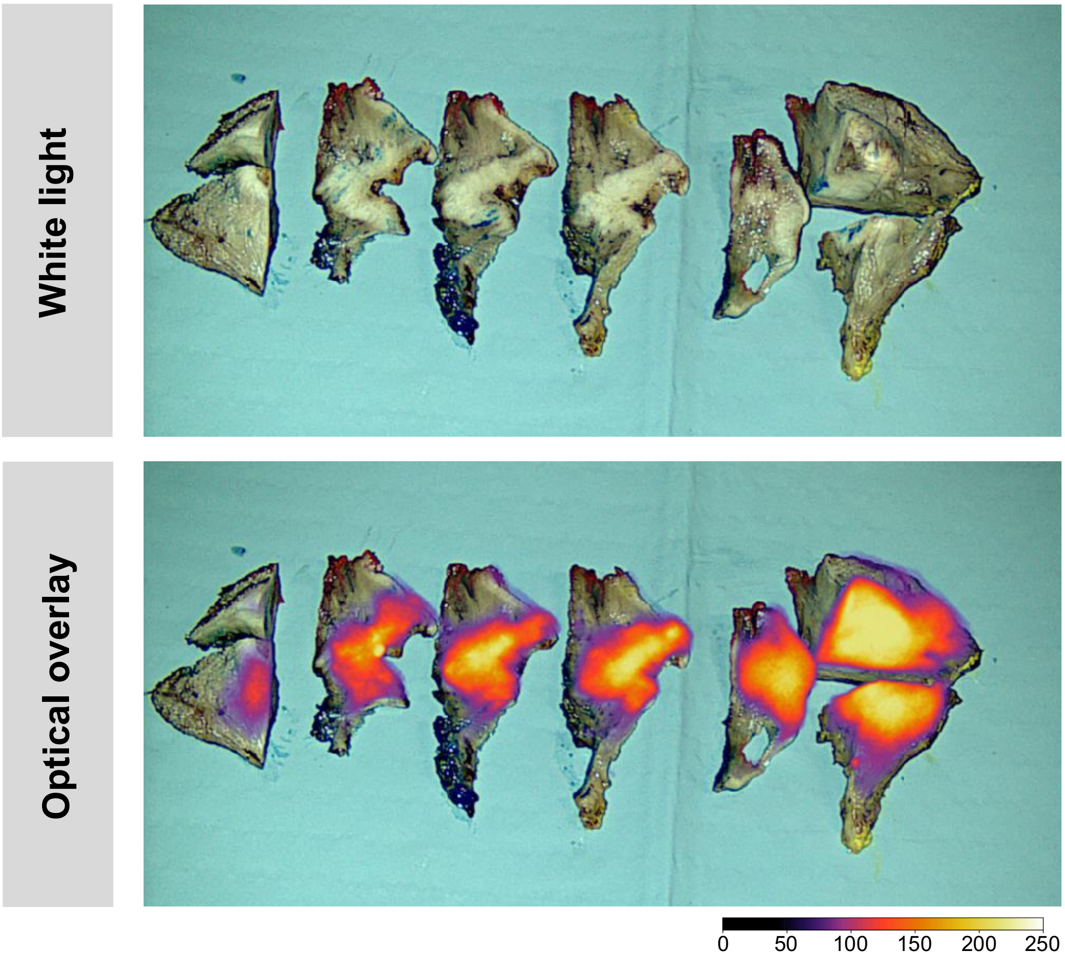

Table S1: Treatment-Emergent Adverse Events (TEAEs) registered during the trial

Adverse Event	Grade 1	Grade 2	Grade 3	Total
Alanine aminotransferase increased			1	1
Alkaline phosphatase increased	1			1
Aspartate aminotransferase increased	2		1	3
Blood bilirubin increased	5	2		7
Creatinine increased	2			2
Dysgeusia (salty taste)	1			1
GGT increased			1	1
Hyperglycemia	2			2
Hypertension		2		2
Hypoalbuminemia	6	4		10
Hyponatremia	1			1
Hypotension	1			1
Hypothermia	3			3
Pneumonia		1		1
**Total**	**24**	**9**	**3**	**36**

Table S2: TEAEs by Primary System Organ Class and Preferred Term

No. subjects (%) / No. events	FG001 36 mg (N=4)	FG001 16 mg (N=8)	FG001 4 mg (N=5)	Total (N=17)
**TEAE**	4 (100.0%) / 14	8 (100.0%) / 15	4 (80.0%) / 7	16 (94.1%) / 36
**METABOLISM AND NUTRITION DISORDERS**	1 (25.0%) / 2	5 (62.5%) / 6	4 (80.0%) / 5	10 (58.8%) / 13
**Hypalbuminaemia**	1 (25.0%) / 1	5 (62.5%) / 5	4 (80.0%) / 4	10 (58.8%) / 10
**Hyperglycaemia**	1 (25.0%) / 1	1 (12.5%) / 1	0 (0.0%) / 0	2 (11.8%) / 2
**Hyponatraemia**	0 (0.0%) / 0	0 (0.0%) / 0	1 (20.0%) / 1	1 (5.9%) / 1
**INVESTIGATIONS**	4 (100.0%) / 9	3 (37.5%) / 4	2 (40.0%) / 2	9 (52.9%) / 15
**Blood bilirubin increased**	4 (100.0%) / 4	2 (25.0%) / 2	1 (20.0%) / 1	7 (41.2%) / 7
**AST increased**	1 (25.0%) / 1	1 (12.5%) / 1	1 (20.0%) / 1	3 (17.6%) / 3
**Blood creatinine increased**	1 (25.0%) / 1	1 (12.5%) / 1	0 (0.0%) / 0	2 (11.8%) / 2
**ALT increased**	1 (25.0%) / 1	0 (0.0%) / 0	0 (0.0%) / 0	1 (5.9%) / 1
**Blood ALP increased**	1 (25.0%) / 1	0 (0.0%) / 0	0 (0.0%) / 0	1 (5.9%) / 1
**GGT increased**	1 (25.0%) / 1	0 (0.0%) / 0	0 (0.0%) / 0	1 (5.9%) / 1
**VASCULAR DISORDERS**	1 (25.0%) / 1	2 (25.0%) / 2	0 (0.0%) / 0	3 (17.6%) / 3
**Hypertension**	1 (25.0%) / 1	1 (12.5%) / 1	0 (0.0%) / 0	2 (11.8%) / 2
**Hypotension**	0 (0.0%) / 0	1 (12.5%) / 1	0 (0.0%) / 0	1 (5.9%) / 1
**GENERAL DISORDERS AND ADMINISTRATION SITE CONDITIONS**	1 (25.0%) / 2	1 (12.5%) / 1	0 (0.0%) / 0	2 (11.8%) / 3
**Hypothermia**	1 (25.0%) / 2	1 (12.5%) / 1	0 (0.0%) / 0	2 (11.8%) / 3
**INFECTIONS AND INFESTATIONS**	0 (0.0%) / 0	1 (12.5%) / 1	0 (0.0%) / 0	1 (5.9%) / 1
**Pneumonia**	0 (0.0%) / 0	1 (12.5%) / 1	0 (0.0%) / 0	1 (5.9%) / 1
**NERVOUS SYSTEM DISORDERS**	0 (0.0%) / 0	1 (12.5%) / 1	0 (0.0%) / 0	1 (5.9%) / 1
**Dysgeusia**	0 (0.0%) / 0	1 (12.5%) / 1	0 (0.0%) / 0	1 (5.9%) / 1

ALP, Alkaline Phosphatase; ALT, Alanine Aminotransferase; AST, Aspartate Aminotransferase; GGT, Gamma-Glutamyl Transferase, PT, Preferred Term; SOC, System Organ Class.
